# Effects of Microstructure on Electrode Properties of Nanosheet-Derived H_x_(Ni_1/3_Co_1/3_Mn_1/3_)O_2_ for Electrochemical Capacitors

**DOI:** 10.3390/nano3020204

**Published:** 2013-03-25

**Authors:** Masato Yano, Shinya Suzuki, Masaru Miyayama, Masataka Ohgaki

**Affiliations:** 1 Research Center for Advanced Science and Technology, The University of Tokyo, 4-6-1 Komaba, Meguro-ku, Tokyo 153-8904, Japan; E-Mails: sin@crm.rcast.u-tokyo.ac.jp (S.S.); miyayama@rcast.u-tokyo.ac.jp (M.M.); 2 Japan Science and Technology Agency, CREST, 5 Sanbancho, Chiyoda-ku, Tokyo 102-0075, Japan; 3 Yokohama Laboratory, Hitachi High-Tech Science Corporation, 1-18-2 Hakusan, Midori-ku, Yokohama, Kanagawa 226-0006, Japan; E-Mail: ogaki-masataka@hhs.hitachi-hitec.com

**Keywords:** nanosheets, H*_x_*(Ni, Co, Mn)O_2_, layer-structured materials, electrochemical capacitors, energy storage devices

## Abstract

Nanosheet-derived H_x_(Ni_1/3_Co_1/3_Mn_1/3_)O_2_ was prepared by restacking (Ni_1/3_Co_1/3_Mn_1/3_)O_2_ nanosheets with large or small lateral sizes and their electrochemical properties in a 1 M KOH aqueous solution; microstructural factors were compared with those of bulk H_x_(Ni_1/3_Co_1/3_Mn_1/3_)O_2_ (HNCM). The electrodes composed of small nanosheets exhibited very large capacitances of 1241 F·g^−1^ (395 mAh·g^−1^) at a current density of 50 mA·g^−1^, and 430 F·g^−1^ (100 mAh·g^−1^) at a large current density of 1000 mA·g^−1^. These large capacitances resulted from a heterogeneous layer structure with a large surface area and pore volume. The electrodes of large nanosheets, with a strongly interconnected microstructure and a surface area slightly larger than that of HNCM, exhibited good cycle stability and capacitances larger than that of HNCM. Microstructural control through the restacking of (Ni_1/3_Co_1/3_Mn_1/3_)O_2_ nanosheets improved the electrochemical properties of H*_x_*(Ni, Co, Mn)O_2_.

## 1. Introduction

The demand for energy storage devices with high power density, high energy density, and high safety, especially for use in power tools and electric hybrid vehicles, is increasing. Electrochemical capacitors have attracted considerable attention as one such type of device because of their high power density, long cycle life, and relatively high energy density [1,2,3]. Moreover, these capacitors are expected to have a high level of safety, especially those that use aqueous solution electrolytes instead of organic electrolytes.

In electrochemical capacitors, electric energy is stored by means of a pseudocapacitive process based on Faradaic redox reactions near the surface of active materials, as well as a capacitive process through charge separation at the electrode/solution interface. Ruthenium oxide has been studied as a cathode material for electrochemical capacitors because of its high electronic conductivity and large capacitance of up to 1580 F·g^−1^ (in very thin films) [4,5,6,7,8]. However, ruthenium oxide is not naturally abundant, and various metal oxides such as V_2_O_5_, MoO_3_, and MnO_2_ have been studied as alternate electrode materials [9,10,11,12,13,14,15,16,17,18,19,20,21,22]. Manganese and vanadium oxides are expected to have large capacitances because their cations take several oxidation states within the potential window of an aqueous solution. In the case of manganese oxides, it is advantageous to use strong basic solutions in which MnO_2_ and Mn(OH)_2_ can exist in solid form [23,24]. However, most Mn-containing compounds exhibit poor cyclic stabilities in basic solutions because of an irreversible phase transformation into spinel-type Mn_3_O_4_ [20,22,25].

Layer-structured Li(Ni_1/3_Co_1/3_Mn_1/3_)O_2_ is a promising cathode material for Li–ion batteries [26,27,28,29,30,31,32,33,34]. Excellent electrochemical properties have been reported for this material, such as large capacities of approximately 150–190 mAh·g^−1^ (284–360 F·g^−1^, 2.5–4.4 V *vs.* Li/Li^+^) with high cycle stabilities and improved electric conductivities achieved through Co-doping [26,27]. We focused on (Ni, Co, Mn) oxides as electrode materials for electrical capacitors, and reported that protonated H_x_(Ni_1/3_Co_1/3_Mn_1/3_)O_2_ (HNCM) has a large capacitance of 400–720 F·g^−1^, high rate capability, and good cycle stability even in a basic KOH aqueous solution [35]. The large capacitance was confirmed to be due to Faradaic redox reactions of component metal ions.

Nanosheets are obtained by delaminating layered materials such as layer-structured compounds into one or more layers [36,37,38,39,40,41]. The thickness of nanosheets is on the nanometer scale, and their lateral dimensions are on the submicrometer to micrometer scale. They are obtained in a colloidal suspension, and their restacking results in a specific surface area much larger than that of conventional particles/powders [6,19,20,36,37]. It has been reported that nanosheet-derived materials exhibit excellent properties as cathode materials for Li–ion batteries [36,37] and electrochemical capacitors [6,19,20]. Because of its low ion diffusion resistance, nanosheet-restacked MnO_2_ exhibits a larger capacitance (173 F·g^−1^) than bulk materials do at high current densities [19].

In the present study, we attempted to improve the electrode properties of layer-structured HNCM by controlling the electrode microstructure through the restacking of (Ni_1/3_Co_1/3_Mn_1/3_)O_2_ nanosheets. (Ni_1/3_Co_1/3_Mn_1/3_)O_2_ nanosheets were obtained by the exfoliation of HNCM. Nanosheet-restacked materials were obtained by restacking the (Ni_1/3_Co_1/3_Mn_1/3_)O_2_ nanosheets with different lateral sizes by using an HCl aqueous solution. HNCM and nanosheet-restacked materials were evaluated by various methods in order to investigate their electrochemical properties and the effects of microstructural changes on the electrode properties.

## 2. Results and Discussion

### 2.1. Microstructures

#### 2.1.1. AFM Observation of Nanosheets

(Ni_1/3_Co_1/3_Mn_1/3_)O_2_ nanosheets were obtained by the exfoliation of layer-structured HNCM [41]. Layer-structured Li(Ni_1/3_Co_1/3_Mn_1/3_)O_2_ (LNCM) was synthesized via a co-precipitation method [28]. A colloidal suspension of (Ni_1/3_Co_1/3_Mn_1/3_)O_2_ nanosheets with small and large lateral sizes (small- and large-NS, respectively) were obtained by multistep centrifugation at different rotation speeds as reported previously [21].

**Figure 1:**
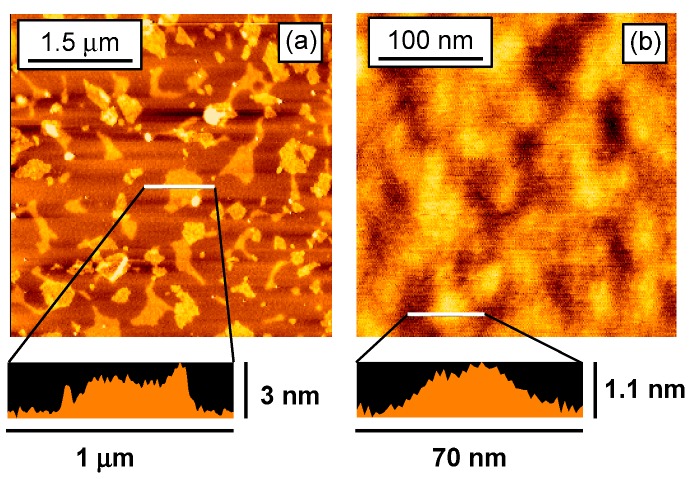
AFM images of (**a**) large-NS and (**b**) small-NS.

Nanosheet sizes were confirmed by atomic force microscopy (AFM) observations. Figure 1 shows the AFM images of the obtained nanosheets deposited on a mica substrate. Large- and small-NS had widths of 50–600 and 50–100 nm, respectively, and thicknesses of 2–3 and 1–2 nm, respectively.

#### 2.1.2. XRD Measurements of Nanosheet-Restacked Materials

Small-NS-restacked materials (as-prepared S-NS-NCM) and large-NS-restacked materials (L-NS-NCM) were obtained by reacting the (Ni_1/3_Co_1/3_Mn_1/3_)O_2_ nanosheet colloidal suspensions with a 1 M HCl aqueous solution. S-NS-NCM was obtained by ball milling of as-prepared S-NS-NCM to reduce its particle size. 

**Figure 2:**
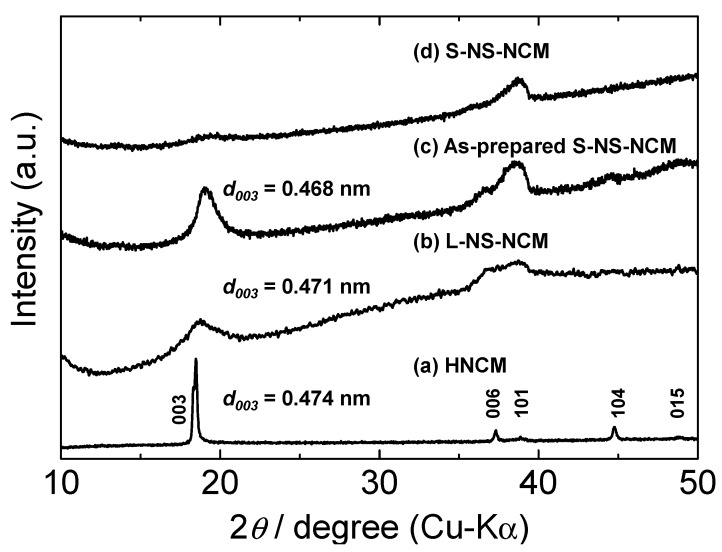
XRD patterns for (**a**) HNCM; (**b**) L-NS-NCM; (**c**) as-prepared S-NS-NCM; and (**d**) S-NS-NCM.

The crystal structures of the particles were confirmed by performing X-ray diffraction (XRD) analysis. Figure 2 shows the XRD patterns for HNCM, L-NS-NCM, as-prepared S-NS-NCM, and S-NS-NCM. The XRD pattern for HNCM exhibited sharp diffraction peaks corresponding to a layered structure similar to that of LNCM. This indicates that HNCM had a regularly stacked layer structure similar to that of LNCM. On the other hand, the XRD pattern for L-NS-NCM and as-prepared S-NS-NCM exhibited broad diffraction peaks. Furthermore, in the case of S-NS-NCM, the diffraction peaks corresponding to the layered structure were quite weak. These results suggest that nanosheet-restacked materials, especially S-NS-NCM, had irregularly stacked layered microstructures.

#### 2.1.3. SEM Observation of Nanosheet-Restacked Materials

**Figure 3:**
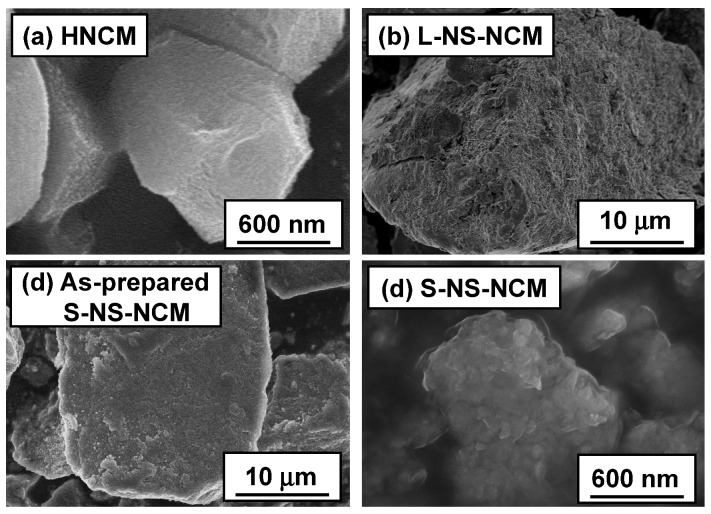
SEM images of (**a**) HNCM; (**b**) L-NS-NCM; (**c**) as-prepared S-NS-NCM; and (**d**) S-NS-NCM.

The morphologies of the particles were observed by scanning electron microscopy (SEM). Figure 3 shows the SEM images of HNCM, L-NS-NCM, as-prepared S-NS-NCM, and S-NS-NCM. HNCM was made up of polygonal particles with sizes ranging from 500 nm to 2 μm. L-NS-NCM contained large particles of approximately 1–20 μm in size, and large-NS stacked on the surface of these particles. As-prepared S-NS-NCM contained large particles approximately 3–50 μm in size, which were composed of irregularly agglomerated small nanosheets of the size 50–100 nm. The particle size of ball-milled S-NS-NCM was reduced to 300 nm to 3 μm, which was almost equal to that of HNCM.

**Figure 4:**
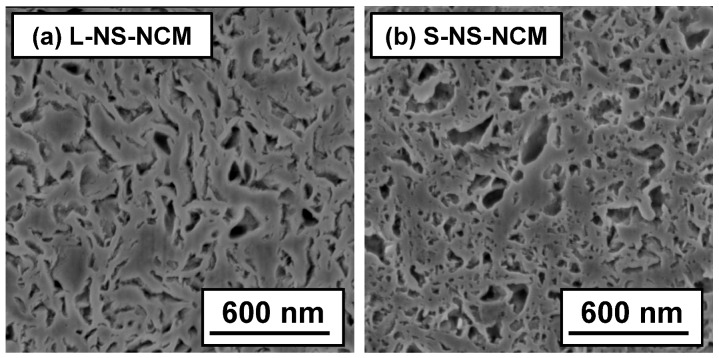
Cross-sectional SEM images of (**a**) L-NS-NCM and (**b**) S-NS-NCM.

The cross-sections of L- and S-NS-NCM particles were also observed by SEM. The cross-sections were prepared by the focused ion beam method. Figure 4 shows the cross-sectional SEM images of L- and S-NS-NCM. Both S- and L-NS-NCM contained numerous pores. In the case of L-NS-NCM, curved large-NS layers were stacked over each other and were strongly interconnected. Moreover, L-NS-NCM maintained a layered structure in the stacked parts. On the other hand, S-NS-NCM had an irregularly stacked layered microstructure made up of small-NS. The interconnection between small-NS layers in S-NS-NCM seemed relatively weaker than that between large-NS layers in L-NS-NCM.

#### 2.1.4. BET Analysis

Table 1 lists the particle size, specific surface area, and pore volume of HNCM, L-NS-NCM, as-prepared S-NS-NCM, and S-NS-NCM. L-NS-NCM had a specific surface area and pore volume of 8 m^2^·g^−^^1^ and 0.02 cm^3^·g^−1^, respectively. These values were almost equal to those for HNCM. On the other hand, as-prepared S-NS-NCM and S-NS-NCM had large specific surface areas of 80 and 56 m^2^·g^−^^1^, respectively, and large pore volumes of 0.22 and 0.14 cm^3^·g^−1^, respectively. The surface areas and pore volumes were increased considerably when small-NS was used. 

**Table 1:** Particle size, specific surface area, and pore volume of HNCM, L-NS-NCM, as-prepared S-NS-NCM, and S-NS-NCM.

Electrodes	Particle size (μm)	Specific surface area (m^2^ g^−1^)	Pore volume (cm^3^·g^−1^)
HNCM	0.5–2	6	0.02
L-NS-NCM	1–20	8	0.02
As-prepared S-NS-NCM	3–50	80	0.22
S-NS-NCM	0.5–3	56	0.14

#### 2.1.5. Summary of Microstructures

The difference in the microstructures of the various samples can be clearly observed from the results of XRD analysis, SEM observations, and BET analysis. HNCM had a homogeneous layered microstructure, while S-NS-NCM had an irregularly stacked layered microstructure. L-NS-NCM had an intermediate structure. L-NS-NCM had a small surface area and pore volume comparable to the surface area and pore volume of HNCM, probably because of its large particle size and surface covered with large-NS. On the other hand, S-NS-NCM had a heterogeneous microstructure with a large surface area and pore volume due to the heterogeneous layered microstructure consisting of small-NS.

### 2.2. Electrochemical Properties

The electrochemical properties of HNCM and NS-NCM were investigated using cyclic voltammetry (CV) tests, constant current charge/discharge tests, and AC impedance measurements. The general reaction mechanisms are first discussed, followed by the experimental results for the electrodes.

#### 2.2.1. General Charge Storage Mechanisms

In electrochemical capacitors, charge is stored via both non-Faradaic capacitive processes and Faradaic pseudocapacitive processes. In a non-Faradaic process, charge is stored rapidly by an electric double layer at the surface of the electrode, which usually has a small charge density of 16–50 μC·cm^−2^. In the case of a Faradaic process for metal dioxide (MeO_2_) electrodes, two mechanisms were proposed by Toupin *et al.* [13].

The first is the adsorption of an alkali metal cation (C^+^) such as K^+^, Na^+^, or Li^+^ in the electrolyte on the surface of the active material.

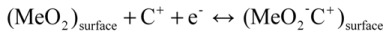
(1)


The second is the insertion of protons (H^+^) or alkali metal cations (C^+^) such as K^+^, Na^+^, or Li^+^ into the lattice of the materials.


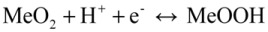
(2)
or

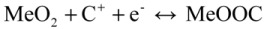
(3)


Both the aforementioned mechanisms are based on a redox reaction between Me^3+^ and Me^4+^. For an alkaline solution system, the following two-step process has been proposed for proton insertion into MeO_2_ [42,43,44]. In the first step, an electron from the external circuit is inserted into MeO_2_ to reduce Me^4+^ to Me^3+^. In the next step, in order to maintain the charge balance, a water molecule present at the MeO_2_/electrolyte interface is decomposed into a proton, which is inserted into the lattice of MeO_2_, and an OH^−^ ion, which diffuses from the interface into the electrolyte.



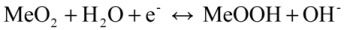
(4)


Me^3+^ is reduced to Me^2+^ in the same way.

#### 2.2.2. Cyclic Voltammograms

**Figure 5:**
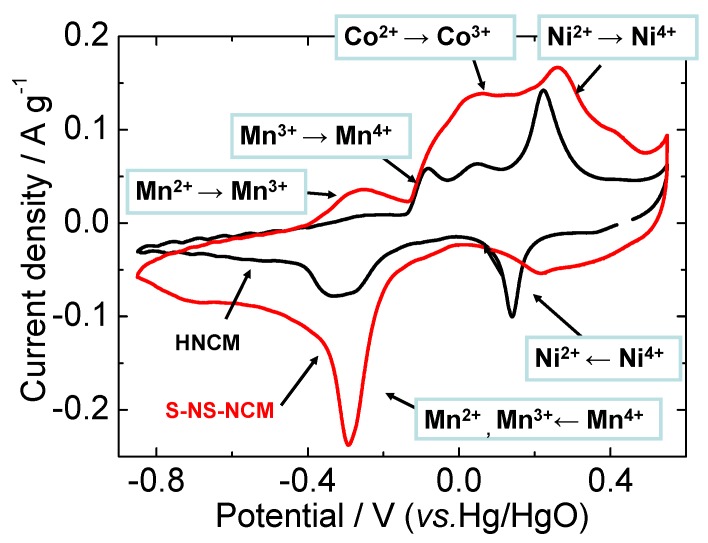
Cyclic voltammograms of HNCM and S-NS-NCM at a sweep rate of 0.1 mV·s^−1^. The potential range was −0.85–0.55 V *vs.* Hg/HgO.

Figure 5 shows cyclic voltammograms (CVs) of HNCM and S-NS-NCM in a 1 M KOH aqueous solution recorded at a sweep rate of 0.1 mV·s^−1^. The redox peaks were observed at almost equal potentials for both HNCM and S-NS-NCM, although S-NS-NCM exhibited larger and broader redox current peaks than HNCM did. This suggests a decrease in the reaction resistance of S-NS-NCM. As reported in our previous study, in which XANES analysis was used for bulk HNCM [35], the reaction of HNCM in an alkaline aqueous system is considered to be a redox reaction between H_2/3_(Ni^4+^_1/3_Co^3+^_1/3_Mn^4+^_1/3_)O_2_ and (Ni^2+^_1/3_Co^2+^_1/3_Mn^2+^_1/3_)(OH)_2_ via reversible insertion/extraction of H^+^. On the basis of the reported redox potential of each ion, the redox currents can be assigned to Ni^4+^/Ni^2+^, Co^3+^/Co^2+^, and Mn^4+^/Mn^2+^ redox reactions [25,45,46], as shown in Figure 5. The reduction current for Co^3+^ to Co^2+^ was not clearly observed. The reason for this can be that Co^3+^/Co^2+^and Mn^4+^/Mn^2+^ reductions occurred simultaneously at the Mn^4+^/Mn^2+^ reduction potential, as reported in the previous study [35]. Only Ni^4+^/Ni^2+^ and Co^4+^/Co^3+^ redox reactions have been reported in previous investigations of the Li insertion/extraction reactions occurring in LNCM in organic electrolyte systems [29,30,31], and Co^3+^/Co^2+^ and Mn^4+^/Mn^2+^ redox reactions observed in the CVs here have never been reported. Co^3+^/Co^2+^ and Mn^4+^/Mn^2+^ redox reactions are thus unique to HNCM in an aqueous solution.

#### 2.2.3. Charge/Discharge Curves

**Figure 6:**
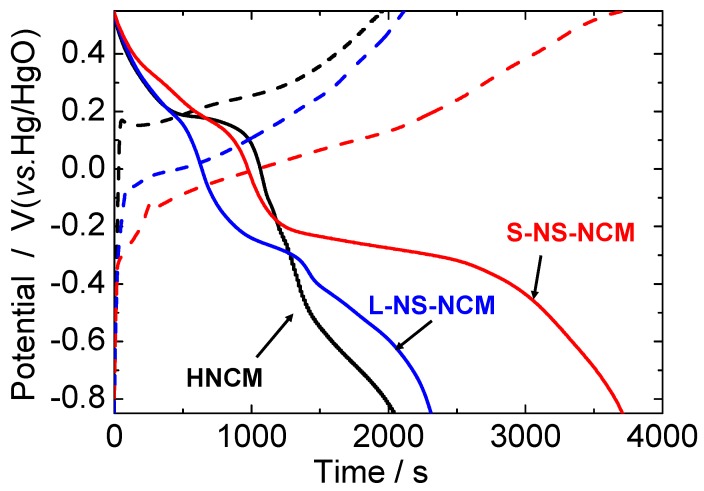
Charge/discharge curves for HNCM, L-NS-NCM, and S-NS-NCM at a current density of 300 mA·g^−1^. The potential range was −0.85–0.55 V *vs.* Hg/HgO.

Figure 6 shows the charge and discharge curves for HNCM, L-NS-NCM, and S-NS-NCM at a current density of 300 mA·g^−1^. In the second cycle, L-NS-NCM and S-NS-NCM showed enhanced capacitances. Two plateaus were observed in the discharge curves. The higher plateau seemed to correspond to the redox reaction of Ni^4+^/Ni^2+^, and the lower plateau seemed to correspond to the reactions of Mn^4+^/Mn^2+^ and Co^3+^/Co^2+^. The differences between the plateau potentials of the charge and discharge curves decreased for L- and S-NS-NCM, which suggested a decrease in the reaction resistance of the nanosheet-derived electrodes.

#### 2.2.4. Cycle Capabilities

**Figure 7:**
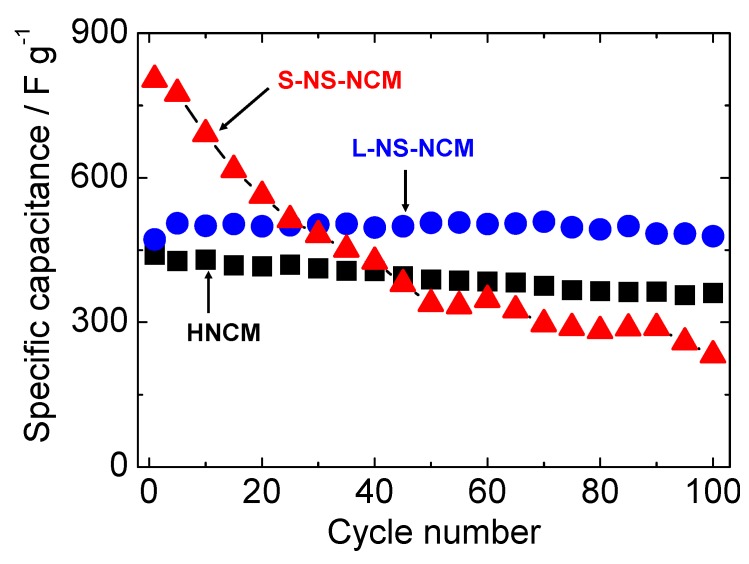
Cycle stabilities of HNCM, L-NS-NCM, and S-NS-NCM at a current density of 300 mA·g^−1^. The potential range was −0.85–0.55 V *vs.* Hg/HgO.

**Figure 8:**
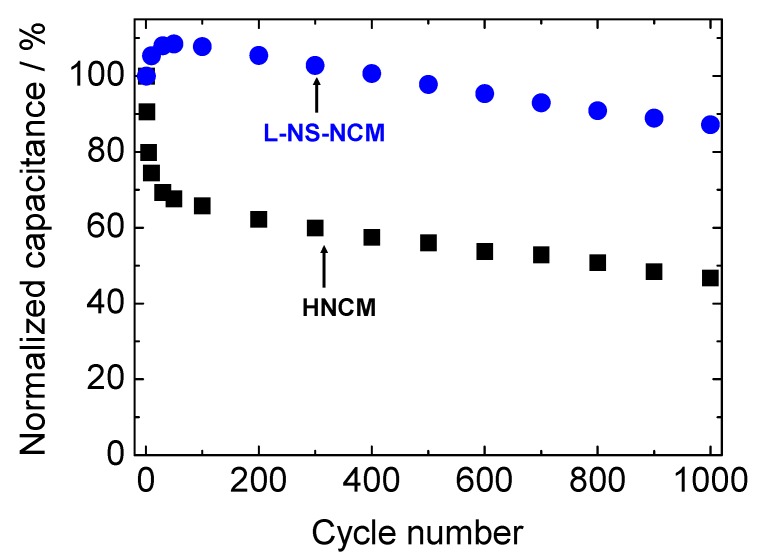
Normalized cycle stabilities of HNCM and L-NS-NCM at a current density of 1000 mA g^−1^. The potential range was −0.6–0.55 V *vs.* Hg/HgO.

Figure 7 shows the cycle performances of HNCM, L-NS-NCM, and S-NS-NCM at a current density of 300 mA·g^−1^. The cycle measurement for HNCM was conducted using the electrode after CV measurement. HNCM and L-NS-NCM exhibited good cycle stabilities and similar capacitances of 441 and 472 F·g^−1^, respectively, in the first cycle, and maintained capacitances of 361 and 479 F·g^−1^ even in the 100th cycle. On the other hand, S-NS-NCM exhibited a large capacitance of 804 F·g^−1^ in the first cycle; however, the capacitance decreased rapidly during the first 50 cycles. The cycle performances of HNCM and L-NS-NCM for longer cycles were measured using new electrodes. Figure 8 shows the normalized cycle performances of HNCM and L-NS-NCM at a current density of 1000 mA·g^−1^. HNCM exhibited a large capacitance of 307 F·g^−1^ in the first cycle; however, the capacitance decreased rapidly to approximately 200 F·g^−1^ during the first 10 cycles but this value was maintained. On the other hand, L-NS-NCM exhibited good cycle performance and a capacitance of 218 F·g^−1^ (87% of that in the first cycle) was maintained even in the 1000th cycle. The difference in the cycle performances of these two compounds probably resulted from the difference in their microstructures. The decay in the capacitances of HNCM and S-NS-NCM seemed to result from microstructural changes in their layered structure during charge/discharge cycles. The original microstructures may have been damaged by lattice volume changes or configuration (rotation, slide) changes in nanosheets during charge/discharge cycles. These effects were remarkable in the case of S-NS-NCM. Such microstructural changes led to blocking of electric/ionic conduction or loss of surface area, resulting in increased reaction resistances. On the other hand, the microstructure of L-NS-NCM was seemingly preserved during charge/discharge cycles, probably because its strongly interconnected layered structure with numerous pores composed of large-NS reduced the physical stress on the particles during charge/discharge cycles. Thus, the excellent cycle stability of L-NS-NCM seemed to have resulted from its strongly interconnected layered structure composed of large-NS.

#### 2.2.5. Rate Capabilities

**Figure 9:**
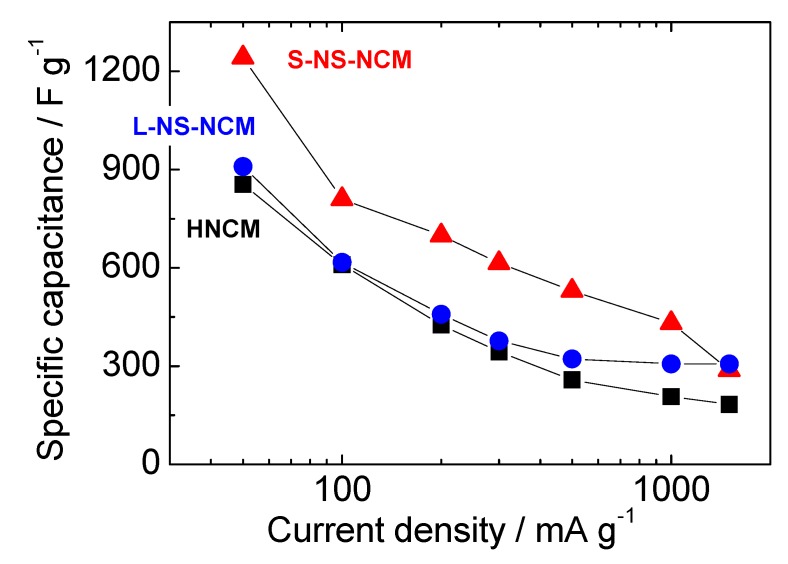
Rate capabilities of HNCM and S-NS-NCM. The potential range was −0.6–0.55 V *vs.* Hg/HgO.

Figure 9 shows the rate capabilities of HNCM and S-NS-NCM. NS-NCM electrodes exhibited larger capacitances than HNCM did at all current densities. L-NS-NCM exhibited slightly larger capacitances than HNCM did in spite of its large particle size. S-NS-NCM exhibited a large capacitance of 1241 F·g^−1^ (395 mAh·g^−1^) at a current density of 50 mA·g^−1^, and 430 F·g^−1^ (100 mAh·g^−1^) at a large current density of 1000 mA·g^−1^. This large capacitance of 1241 F·g^−1^ corresponds to approximately 80% of the theoretical maximum capacitance of the redox reactions of Ni^4+^/Ni^2+^, Co^3+^/Co^2+^, and Mn^4+^/Mn^2+^ (~1250 F·g^−^^1^, 400 mAh·g^−1^ ). This capacitance is larger than that reported for RuO_2_/C composites (980 F·g^−1^) [8], and a capacitance larger than this has not been reported for powder (except for very thin films) electrode materials for electrochemical capacitors [4,5,6,7,8,9,10,11,12,13,14,15,16,17,18,19,20,21,22].

#### 2.2.6. AC Impedance Measurements

**Figure 10:**
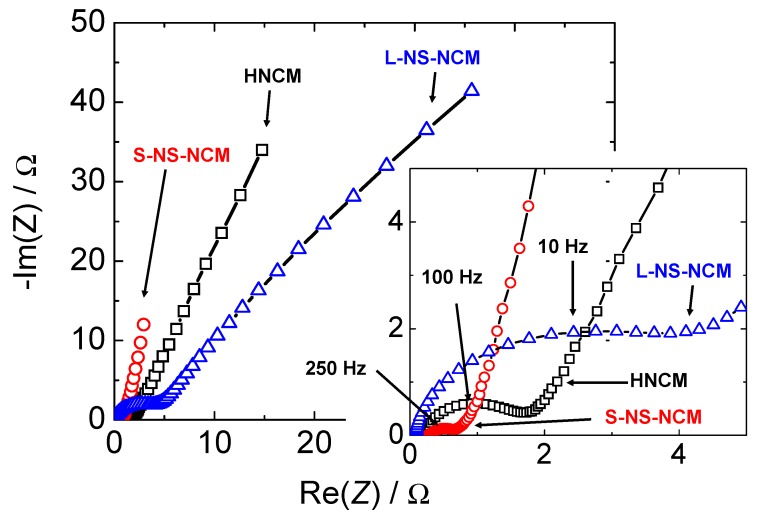
AC-impedance plots for HNCM, L-NS-NCM, and S-NS-NCM.

**Figure 11:**
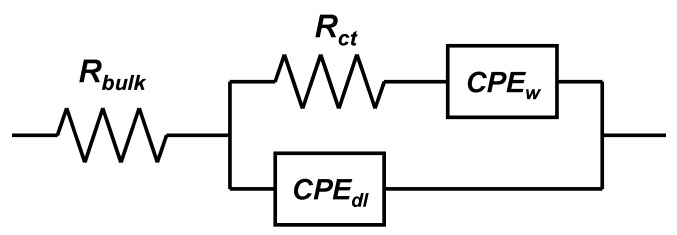
Equivalent circuit used for curve fitting.

AC impedance measurements were conducted in order to confirm the difference in the reaction resistances of these electrodes. Figure 10, Figure 11 show AC impedance plots for the electrodes in the first charge cycle and the equivalent circuit used for curve fitting, respectively. The reaction resistances obtained by curve fitting using an equivalent circuit are listed in Table 2. The real-axis intercept in the high-frequency region corresponds to the bulk resistance *R_bulk_*, which includes the electronic resistance of the active material, contact resistance with the current collector, and electrolyte resistance. The curve in the high-to-medium frequency region corresponds to the charge-transfer reaction *R_ct_*. In this region, an electron from the external circuit is inserted into MeO_2_, and this is followed by the insertion reaction of a proton in order to maintain the charge balance. The straight-line sections with slopes of 45–90° in the low-frequency region correspond to ionic diffusion in the electrode [47,48].

S-NS-NCM had the largest double-layer capacitance; moreover, the double-layer capacitance of L-NS-NCM was slightly larger than that of HNCM. The double-layer capacitance increased with the specific surface area. The ionic diffusion resistance and charge-transfer resistance were the largest and smallest for L-NS-NCM and S-NS-NCM, respectively. The differences in these resistances are also considered to have resulted from the difference in the microstructures of these electrodes, as discussed in the next section.

**Table 2:** Reaction resistances of HNCM, L-NS-NCM, and S-NS-NCM obtained by curve fitting with an equivalent circuit.

Electrodes	Charge transfer resistance ( )	Double-layer capacitance (m^2^ g^−1^)	Diffusion resistance (qualitative)
HNCM	1.53	2.2 × 10^−^^3^	Intermediate
L-NS-NCM	3.81	2.9 × 10^−^^3^	Large
S-NS-NCM	0.56	5.7 × 10^−^^2^	Small

#### 2.2.7. Discussion

The ionic diffusion process in an electrode involves ionic diffusion in the solid state, as well as in the electrolyte-filled pores. The ionic diffusion resistance obtained from the AC impedance measurements also involves the contributions of these two diffusion processes. The ionic diffusion processes in the solid state and in the pores are largely influenced by particle size and pore volume. Since L-NS-NCM had a large particle size and a small pore volume, its ionic diffusion resistance was more than those of the other electrodes.

HNCM and S-NS-NCM had similar particle sizes but different microstructures. HNCM had a homogeneous layered structure with a small surface area and pore volume, while S-NS-NCM had a heterogeneous layered structure with a large surface area and pore volume. For layer-structured oxides, the diffusion rate of cations as alkaline metal ions is large in parallel and small perpendicular to the layers [49]. Therefore, in a material with a homogeneous layered structure consisting of regularly stacked layers, such as HNCM, a proton has to travel a long distance to reach the inner part of the active material. In contrast, proton diffusion should be smooth in S-NS-NCM through the electrolyte in the pores and inside the nanosheets with small lateral sizes; this could be the reason for the difference in the diffusion resistances of HNCM and S-NS-NCM.

A large surface area, fast transport of ions, and sufficient supply of electrons are important factors to achieve a fast charge-transfer reaction. When an electrode has a large pore volume, enough electronic conductivity, and large specific surface area, the charge-transfer resistance will be small. All the aforementioned conditions are assumed to be present in S-NS-NCM. It had a small charge-transfer resistance because of a heterogeneous layered structure with a large surface area and pore volume.

Accordingly, the large capacitance and high rate capability of S-NS-NCM as compared to the other electrodes can be attributed to the heterogeneous layered structure of S-NS-NCM with a large surface area and pore volume.

The capacitances of L-NS-NCM were larger than that of HNCM in most cases, although the reaction resistances in the 1st charge cycle were larger than those for HNCM. Original microstructures may have been lost or damaged by changes in the cell volume or sliding of the oxide layer during charge/discharge cycles, especially in S-NS-NCM because nanosheets seemed to be weakly interconnected in S-NS-NCM. This microstructural change may have caused physical damage of particles, loss of surface areas, or electric conductive passes, which resulted in increased reaction resistances. On the other hand, the microstructure of L-NS-NCM was seemingly preserved during charge/discharge cycles, probably because its strongly interconnected layered structure with numerous pores composed of large-NS reduced the physical stress on the particles during charge/discharge cycles. Thus, the excellent cycle stability of L-NS-NCM seemed to have resulted from the strongly interconnected layered structure composed of large-NS.

On the basis of these effects, the restacking of (Ni_1/3_Co_1/3_Mn_1/3_)O_2_ nanosheets was found to be an effective method to improve the electrode properties of layer-structured materials for electrochemical capacitors with high rate capability, large specific capacitance, and good cycle stability. The electrode properties of HNCM could be further improved by precise microstructural control achieved through nanosheet restacking. Particles with an intermediate microstructure between those of S- and L-NS-NCM, for which strong interconnection of nanosheets and large surface area co-exist, will have high rate capability, large specific capacitance, and good cycle stability. The rate capability can be improved by fabricating homogeneous nanosheet composites and electronically conductive additives; moreover, the cycle stability can be enhanced by microstructural control such as interlayer modification through the use of different kinds of cations.

## 3. Experimental Section

### 3.1. Material Synthesis

Nanosheets were obtained by the exfoliation of layer-structured HNCM [41]. Layer-structured LNCM was synthesized via a co-precipitation method [28]. Aqueous solutions of NiSO_4_, CoSO_4_, and MnSO_4_ (Ni:Co:Mn = 1:1:1) with a total concentration of 2.0 M were added into a continuously stirred reactor under an Ar atmosphere. Simultaneously, a 2.0 M NaOH aqueous solution as a pH conditioner and an NH_4_OH aqueous solution (metal ion:NH_4_OH = 1:1) as a chelating agent were also separately added into the reactor. The co-precipitated particles were stirred at 80 °C for 12 h. The pH value during co-precipitation was maintained at 12. The obtained (Ni_1_*_/_*_3_Co_1_*_/_*_3_Mn_1_*_/_*_3_)(OH)_2_ particles were filtered, washed, and dried at 80 °C in vacuum for 2 h. The LiOH·H_2_O powders were then mixed and calcined. The mixture was first heated at 480 °C for 5 h in air and was then calcined at 1000 °C for 10 h in air to obtain LNCM powders. To obtain HNCM, the interlayer Li was exchanged for protons by stirring LNCM in a 1 M HCl aqueous solution at room temperature for 5 days, during which time the HCl solution was refreshed daily. A colloidal suspension of (Ni_1/3_Co_1/3_Mn_1/3_)O_2_ nanosheets was obtained by reacting HNCM with tetramethylammonium hydroxide at room temperature for 14 days. A colloidal suspension of small- and large-NS was obtained by multistep centrifugation at different rotation speeds, as reported previously [21]. As-prepared S-NS-NCM and L-NS-NCM were obtained by reacting the (Ni_1/3_Co_1/3_Mn_1/3_)O_2_ nanosheet colloidal suspensions with a 1 M HCl aqueous solution. The particle size of as-prepared S-NS-NCM was reduced by ball milling in ethanol at 200 rpm for 1 h, after which the ethanol was evaporated.

### 3.2. Characterizations

The crystal structures of the samples were confirmed by performing XRD analysis using a D8 ADVANCE diffractometer (Bruker, Billerica, MA, USA). The morphologies of the delaminated nanosheets were observed by AFM performed using a SPS3100 microscope (Seiko Instruments, Chiba, Japan). The particle morphologies were observed by SEM performed using S-4500 and SU-8000 microscopes (Hitachi High-Technologies, Tokyo, Japan). The cross-section processing of the particle samples and the observation were performed by double beam system of the focused ion beam and the SEM (FIB-SEM) system, using XVision210DB (Hitachi High-Tech Science, Tokyo, Japan). The specific surface area and pore volume of the samples were determined by a gas absorption method carried out using a Micromeritics TriStar 3000 analyzer.

### 3.3. Electrochemical Measurements

HNCM and NS-NCM electrodes were prepared by mixing the active material (HNCM or NS-NCM), acetylene black, and PTFE in a weight ratio of 5:5:1, and pressing the mixture onto a Ti mesh under a loading of 5 mg·cm^−2^. Electrochemical measurements were performed using a three-electrode cell with a Hg/HgO electrode as the reference electrode and a Pt mesh as the counter electrode. KOH aqueous solutions with a concentration of 1 M were used as the electrolyte. In addition, galvanostatic charge/discharge tests were carried out using a Solartron 1470E Cell-Test system (Solartron Analytical, Hampshire, UK), a HZ-3000 system (Hokuto Denko, Tokyo, Japan), and a VMP3 (BioLogic, Grenoble, France). AC impedance measurements were carried out using the BioLogic VMP3.

## 4. Conclusions

The S-NS-NCM electrode composed of (Ni_1/3_Co_1/3_Mn_1/3_)O_2_ nanosheets with a small lateral size of 50–100 nm exhibited very large capacitance of 1241 F·g^−1^ (395 mAh·g^−1^) at a current density of 50 mA·g^−1^, and 430 F·g^−1^ (100 mAh·g^−1^) at a large current density of 1000 mA·g^−1^ in a 1 M KOH aqueous solution. The heterogeneous layered structure of S-NS-NCM with a large surface area and pore volume contributed to an increase in the reaction surface area and smooth ion diffusion in the electrodes, resulting in a small reaction resistance. Electrodes of large nanosheets with sizes of 50–600 nm and surface areas slightly larger than that of HNCM exhibited good cycle stability, maintaining 218 F·g^−1^ (87% of that in the first cycle) at 1,000 mA·g^−1^ even in the 1000th cycle. Microstructural control through the restacking of (Ni_1/3_Co_1/3_Mn_1/3_)O_2_ nanosheets was found to be effective for improving the electrochemical properties of HNCM.
